# Mediterranean Diet in Developmental Age: A Narrative Review of Current Evidences and Research Gaps

**DOI:** 10.3390/children9060906

**Published:** 2022-06-16

**Authors:** Ilaria Farella, Francesca Miselli, Angelo Campanozzi, Francesca Maria Grosso, Nicola Laforgia, Maria Elisabetta Baldassarre

**Affiliations:** 1 Department of Biomedical Sciences and Human Oncology, Clinica Medica “A. Murri”, University of Bari “Aldo Moro”, 70124 Bari, Italy; 2 Neonatal Intensive Care Unit, University Hospital of Modena, 41125 Modena, Italy; miselli.fnc@gmail.com; 3 Department of Pediatrics, University of Foggia, 71122 Foggia, Italy; angelo.campanozzi@unifg.it; 4 Postgraduate School of Public Health, University of Milan, 20136 Milan, Italy; francy92@gmail.com; 5 Department of Interdisciplinary Medicine, University of Bari “Aldo Moro”, 70124 Bari, Italy; nicola.laforgia@uniba.it

**Keywords:** Mediterranean diet, weaning, developmental age

## Abstract

Numerous studies in recent decades have shown that Mediterranean diet (MD) can reduce the risk of developing obesity in pediatric patients. The current narrative review summarizes recent evidence regarding the impact of MD across the different stages of child development, starting from fetal development, analyzing breastfeeding and weaning, through childhood up to adolescence, highlighting the gaps in knowledge for each age group. A literature search covering evidence published between 1 January 2000 and 1 March 2022 and concerning children only was conducted using multiple keywords and standardized terminology in PubMed database. A lack of scientific evidence about MD adherence concerns the age group undergoing weaning, thus between 6 months and one year of life. In the other age groups, adherence to MD and its beneficial effects in terms of obesity prevention has been extensively investigated, however, there are still few studies that correlate this dietary style with the incidence of non-communicable diseases. Furthermore, research on multi-intervention strategy should be implemented, especially regarding the role of education of children and families in taking up this healthy dietary style.

## 1. Introduction

The Mediterranean diet (MD) is a term given to a dietary regimen characterized by a predominant consumption of vegetables, fruit, legumes, whole grain cereals, nuts seeds, olive oil, low intake of red meat, low to moderate intake of fish and poultry, moderate but steady intake of dairy products, and a moderate wine drinking habit [1]. The MD is considered as one of the most balanced dietary patterns worldwide, as it is particularly rich of plant-based proteins, low glycemic index carbohydrates, monounsaturated fatty acids, dietary fibres, and antioxidants. Unfortunately, the western diet (rich in saturated fats, refined carbohydrates, and salt) is registering a rising trend in popularity among children, thus resulting in the increase in incidence of chronic non-communicable diseases, especially those related to obesity [2]. 

Children will go through different stages of development, during the process of growth and varied nutritional needs that will have to be met to sustain a constantly changing body. Moreover, every child will also withstand several social influences (from parents, their school, and ultimately from their peers). The “first thousand days of life” (including nine months of intrauterine life, 270 days, and the first two years of a child’s life, 730 days) do affect the child’s future health greatly; it is a very vulnerable period due to rapid growth, high nutritional needs, and complete dependence on others for feeding and social interactions [3,4]. It is noteworthy that many interventions have aimed to correct dietary patterns during childhood have significantly improved children’s weight trajectory, although effects tended to diminish over time [5]. Important changes have occurred over the preschool period and school years, such as progressively less control being exterted by parental figures and the gradual acceptance of kindergarten and school’s canteens as food providers [6]. Adolescence is marked by changes in hormonal balance, in lifestyle, and diet that become increasingly influenced by peers [7]. The aim of this narrative review is to synthesize current evidence regarding MD diffusion and impact in the developmental age for each age group; from pregnancy, the nursing and weaning period, preschool age, primary school age, and adolescence. Furthermore, research gaps for each age group are highlighted (Table S1) with the aim of providing ideas for future research and implementing interventions to foster healthy habits, which if acquired early, are likely to persist into adulthood [8].

## 2. Material and Methods

A search was performed on PubMed (nih.gov, accessed on 11 April 2022), screening for international papers (both observational, interventional studies) concerning the effects of MD on newborns, infants, children, and adolescents. The search was not restricted by language. We used standardized terminology in Pubmed database, combining MeSH and free text terms for “child health” or “offspring” or “newborn” or “neonate” or “child” or “baby” or “gestation” or “pregnancy” or “pregnant woman” or “perinatal period” or “prenatal exposure” or “prenatal” or “infant” or “child” or “adolescence” or “pediatric” or “weaning” and “Mediterranean diet”or “fruit” or “vegetable” or “legume” or “nut” or “olive oil” or “evoo” or “oily fish” or “seafood” or “tomato”. All titles/abstracts identified in the electronic databases were screened by two authors (I.F., M.E.B.) independently. The search covered articles published between 1 January 2000 and 1 March 2022.

## 3. MD and Pregnancy

The Developmental Origin of Health and Disease theory assumes that the origins of lifestyle-related diseases are rooted in embryonic, fetal, and neonatal ages [9]. Maternal nutrition during pregnancy has a pivotal influence on the in-uterus environment, therefore on the fetal growth and development [8,9, 10]. The adherence to the MD in pregnant women is still not satisfactory; several European cohort studies reported a low to moderate adherence [10]. Nevertheless, the highest adherence was observed among women with a higher income, education, and higher levels of physical activity [11,12,13]. The effect of MD during pregnancy on the incidence of gestational diabetes, congenital defects such as gastroschisis in offspring, prematurity, and lower birth weight (SGA) is widely recognized [14,15]. However, Biagi et al. point out the need for further randomized control trials to confirm the intermediate-level evidence linking maternal adherence to MD to the birth of a small-for-gestational-age baby, to preterm delivery, fetal growth restriction, and neural tube defects [16]. This research gap was partially filled by a Spanish randomized control trial conducted on three groups of women with high-risk pregnancies who underwent either MD or mindfulness-based stress reduction interventions versus usual care. The study showed that the percentage of newborns with birth weight below the 10th percentile dropped significantly in the groups that received either one or the other intervention [17].

The consumption of olive oil (a cornerstone of the MD) has a role in preventing gestational diabetes and small for gestational age (SGA) newborns and is well demonstrated too. Assaf-Balut et al. showed that an MD regimen with an additional quantity of extra virgin olive oil and pistachios reduces both the incidence of gestational diabetes in women and the incidence of SGA newborns [18]. Martínez-Galiano et al. conducted matched case-control study on 1036 women; they concluded that an intake of olive oil above 5 g/day was associated with a lower risk of SGA [19].

The epigenetic changes during fetal life, influenced by maternal MD uptake, would have long-term beneficial effect on the concentration of serum homocysteinemia and lipoproteins as well as on the development of metabolic syndrome in offspring [20,21].

Regarding atopy in offspring, evidence is contrasting because of the low quality of data coming from current studies [16]. Additional studies are needed to better clarify the role of maternal adherence to the MD on asthma/allergy. However, a large population-based longitudinal study in the UK (including 13,972 singleton or twin children followed up to 7 years of life) found that maternal MD regimen adherence was not associated with asthma nor other allergic outcomes in the offspring, but rather with increased small airway function in childhood (forced expiratory flow at 25–75% of forced vital capacity (FEF25–75%)) [22].

An innovative concept is that nutritional education could start “in utero”; Mennella et al. evidenced how adding flavour to amniotic fluid or breast milk by having mothers drinking carrot juice regularly, could increase infants’ acceptance and enjoyment of similarly flavoured foods during weaning. Ideally, children used to the typical tastes of MD early may naturally prefer this healthy eating style [23].

## 4. Mediterranean Diet, Nursing, and Weaning

Exclusive breastfeeding for 6 months, followed by an alternate regimen of breastfeeding and complementary nutrition until 12 months is strongly recommended to provide the right nourishment to children [24]. The quality of breast milk and the adoption of an adequate weaning diet is a cornerstone in the development of a life-long healthy status of the offspring. To date, although there is growing interest in alternative weaning models [25,26], few studies investigated the role of MD during lactating and weaning times. Gila-Díaz et al. compared a population of lactating women with pregnant and non-lactating-non pregnant women; an interesting finding is that the adherence to MD, assessed with the “Adherence to the Healthy Food Pyramid questionnaire” was lowest in lactating women, while pregnant women had the highest scores [27,28]. In addition, investigators found a positive correlation between maternal age, socioeconomic status, and education and the adherence to MD in lactating and pregnant women [11]. Furthermore, the ECLIPSES study shows interesting results; the diet of Spanish pregnant women deviates from MD (red and processed meat and sweet food exceed recommendations) and progressively worsens from the first trimester of pregnancy to the post-partum period (consumption of fruits, vegetables, cereals is reduced) [12]. During the post-partum period, mothers have lower adherence to MD, so it is important to start a diet re-sensitization program. The diet followed in the post-partum period would also influence the composition of breast milk as demonstrated by the MEDIDIET study [29]; the food frequency questionnaire [30] identified five dietary patterns; “Vitamins, minerals and fibre”, “Proteins and fatty acids with legs”, “Fatty acids with fins”, “Fatty acids with leaves”, “Starch and vegetable proteins”. These patterns correlate with the amounts of fatty acids, in particular ω-3, in milk in 300 healthy Italian breastfeeding mothers, underlining the importance of adequate maternal nutrition during lactation to provide the infant with a balanced milk [29].

Mazzocchi et al. foster MD as an example of a sustainable nutrition model starting from the introduction of solid foods in infancy because it emphasizes the parental role model (home environment, foods provided, feeding practices) as an effective method for improving children’s diet and eating behavior [31].

A very interesting systematic review conducted by Netting et al. showed that, even if no correlation between mothers’ dietary intake and atopic outcome in children are described, eating according to MD during lactation was among the few consistent associations with a lower risk of allergic disease in children [32].

## 5. MD in Preschool Age

Preschool age (1–5 years) is a delicate age group because after one year of age, and thus fully weaned, pediatric visits become less frequent and attention to nutrition is reduced. Child nutrition is mostly the task of caregivers, members of the family and kindergarten rather than expert professionals [6]. The type of diet followed during this age can influence the risk of becoming overweight, obese, or building abdominal obesity later in life [33]. A systematic review reports that a low adherence to MD in preschool children living in the Mediterranean countries of the European Union is associated with a high prevalence of being overweight or obese [6]. In fact, despite adequate consumption of fruits and vegetables, preschool children are often exposed to sugary drinks, snacks, and an excessive intake of proteins and sodium [6]. A study performed on 619 Spanish children highlighted that a higher adherence rate to MD and higher cardiopulmonary fitness (measured with 20 m shuttle run test) are associated with lower waist circumference, an anthropometric parameter widely associated to an increased risk of cardiovascular diseases [34]. It is noteworthy how MD has a protective role against wheezing [35] and acute rhinosinusitis [36], two very frequent causes of pediatrician consultation in preschool age. Furthermore, a higher adherence to MD at 1 year seems to be significantly associated with a lower risk of celiac disease autoimmunity at 6 years [37].

A fundamental role in this age group is played by parents, in fact several studies show a positive correlation between parental education, high socio-economic status parent’s health awareness [38] and a better quality of diet [39]. As demonstrated by Roset-Salla et al., the educational interventions performed on parents once their child was able to sit at the dinner table (1–2 years old) showed a significant increase in adherence of the children to MD, suggesting that intervention strategies should also target this time in life in order to achieve maximum success [40].

## 6. MD in School Children

Children that go to school are usually 6 to 12 years old, while adolescents range from 10 to 19 years old [41], thus, there is often overlapping between the two age groups. Considering that studies in the literature are often conducted on a comprehensive age group ranging from late childhood to adolescence, overlapping studies are also included in this analysis for educational purposes.

Adherence to MD greatly varies in Mediterranean countries for school children, with important differences among European countries, while adherence in non-Mediterranean countries is poorly investigated [42]. The data regarding MD adherence are often expressed in different ways, thus making the comparison between studies difficult. When the results are expressed by a mean of the KIDMED score [43], the MD adherence varies between 3.6 in Greek adolescents to 7.60 in Spanish children. Instead, when the results are expressed as three categories of KIDMED score, a poor adherence ranges from 1.6% in Spanish children to 62.8% in Greek adolescents, average adherence from 28.0% in Greek adolescents to 73.8% in rural Italian adolescents, and good adherence from 4.3% in Greek 10–12 year old subjects to 53.9% in Spanish children [42]. In Italy, the rate of adherence to MD is low in schoolers; only 5.0% of them strictly follows MD. Nevertheless, adherence improves with lunch at school, a finding that emphasizes the role of the school in children’s health [44]. Several studies point out the importance of physical activity and screen time in predicting both the adherence to MD [45,46] and the development of higher general or abdominal adiposity [47]. The acquisition of MD habits in this age range seems to have a direct protective effect on the development of several diseases; asthma and allergic rhinitis in 6–7 years old children [48], severe asthma in 6–7 years old girls [49], gastrointestinal disorders in 6-18 years old children and adolescents [50], arterial hypertension [51] and metabolic syndrome in adults [52].

Educational interventions can be designed so that they are focused both on parents and on schoolers and adolescents. Fernandez-Ruiz et al. demonstrated the effectiveness of a 12-month multidisciplinary program based on individual and group (family) education, cognitive-behavioral therapy, healthy diet, and physical activity on decreasing body mass index and metabolic parameters in overweight and obese children (6–12 years). These positive effects were maintained until 1 year after the end of the intervention, suggesting long-lasting effects [53]. Similar findings have been shown in a Spanish cohort in which a nutritional education program and an intensive lifestyle intervention were able to reduce BMI in 107 children with abdominal obesity [54]. However, as pointed out by Lassale et al., results that investigated the correlation between MD and obesity are controversial; some trials report a beneficial effect of the dietary intervention, whereas others did not. Very few studies include a longitudinal analysis, and the majority of observational and intervention studies in the literature are of low quality [55].

## 7. MD in Adolescents

Adolescence is characterized by physical and psychological changes due to puberty and sexual development [56]. During adolescence, dietary patterns and behaviors are influenced by several factors; parental and peer models, food availability, food preferences, cost, cultural beliefs, mass media, social media, and body acceptance [7]. Adherence to MD of Israeli adolescents has improved from 2003 to 2016 (the percentages with poor, average, and good KIDMED scores were 11.6, 45.3 and 43.1% in 2015–2016, compared to 25.5, 55.2, and 19.3%, respectively, in 2003–2004), as demonstrated by two national health and nutrition surveys conducted in Israeli schools [57]. In Italy, low adherence was observed in 38.6% of teenagers, moderate adherence in 47.4% and high adherence in 14.0%, with the highest adherence in those from the southern regions [58]. In Spain, 49% of adolescents reported following an optimal MD [59]. Balearic Island adolescents had moderate adherence to MD, with a higher adherence observed among consumers of functional foods [60]. In non-Mediterranean countries such as Lithuania, only 14% of adolescents followed MD [61]. Several studies demonstrated a positive correlation between the adherence to MD and physical activity [57,62,63], a correct sleep schedule [64], high socioeconomic status, and having the habit of reading food labels [63]. Diet support sought from family members and trust in school teachers are correlated to better rates of MD adherence [61]. Furthermore, living in a smaller city or rural location [63] is associated with better MD adherence [63]. Instead, long hours spent watching television and videos [65], computer use [63,66] and low parental education were risk factors for lower adherence to MD [60,63,64,67,68]. Adherence to MD is consistently and negatively associated with high percentages of body fat and obesity [65,69,70], whereas it is positively associated with a higher aerobic capacity [71] and cardiovascular endurance [72]. Nevertheless, an optimal adherence to MD may not overcome the deleterious effects of low physical fitness on cardiovascular disease risk in adolescents [73].

The impact of MD on mental health in adolescents has been equally well demonstrated with a positive impact on self-esteem, self-concept, and global life satisfaction [65]. [59,65]. Additionally, MD is associated with adolescents being more active, with better academic performance [59,74], with better elaboration and organization skills, and critical thinking [74]. On the other hand, poor adherence to MD, obesity, and low physical activity levels negatively affect academic performance in boys and girls [69,75].

Arenaza et al. demonstrated the effect of educational programs on adolescents; a 22-weeks family-based healthy lifestyle psychoeducational program positively improved diet quality and health in children with overweight/obesity. Sugar-sweetened beverages avoidance was associated with a hepatic-fat reduction [51].

## 8. Discussion and Conclusions

Despite the cumulative data reporting the possible beneficial effects of MD diet on children, this narrative review, showed that few studies have evaluated the effects of MD on specific age groups [52,53,76]. As we move towards more “tailored” medicine [77], it is important to perform ad hoc studies for each age group to avoid biased generalizations. Studies considering specific age and gender groups at the same time must certainly also be implemented [78].

Adherence to MD is often difficult to compare between the various age groups because of the heterogeneity of indexes and methodologies used to assess it and the multiple definitions existing for MD [79]. Furthermore, selecting the most appropriate diet quality index is complex because only few indexes have been evaluated for validity, reliability, or have been correlated with health outcomes [80]. Adherence to MD varies across countries (Mediterranean and non-Mediterranean) and among different developmental stages of children. Higher rates of adherence to MD are observed during pregnancy and then gradually decrease during lactation [11,12]. Poor adherence is observed during preschool and school age [12,64], but it increases positively during adolescence [58,63,64]. These results are helpful for identifying who is at the highest risk of developing unhealthy eating habits, which should be targeted with nutritional re-educational programs. A lack of evidence concerns weaning; currently, there are no studies that investigate MD adherence in this age group, which is of interest as it is part of the first 1000 days of a child’s life. This gap in knowledge should be filled because the type of diet during weaning can influence the child’s weight trajectory in a preponderant way. There are still very few studies correlating MD in pregnancy and the development of malformations in the unborn fetus, as few are the evidence correlating maternal MD adherence to fetal growth restriction, risk of SGA newborn and preterm delivery. [17]. Currently, there is only one randomized controlled study that implemented an early nutritional intervention with MD supplementation with extra virgin olive oil and pistachios, resulting in a significant reduction in preterm birth rate [18]. This outcome is particularly difficult to evaluate given the different definitions used for preterm, which range from less than 37 weeks to earlier gestational age [17]. Reduced risk of neural tube defects, gastroschisis and MD are related, however, evidence is still lacking [17]. For the correlation between the incidence of asthma and/or allergic diseases due to the heterogeneity of studies [15].

Few studies have investigated MD and the development of non-communicable disease during childhood and adolescence. In spite of the particular attention to allergic-respiratory diseases and obesity [33,34,36,65], there are few reports regarding the correlation between MD and endocrinological, neuropsychological, gastrointestinal diseases, and more generally, with various early-onset autoimmune disorders [38]. Furthermore, although the link between obesity and MD are largely investigated, evidence is scarce regarding the association between MD and maintaining a correct body weight in childhood. More longitudinal and high-quality intervention studies are highly needed to design appropriate programs against the obesity burden in children and adolescents [55]. Another consistent research gap concerns the link between gut-microbiota composition, MD, and obesity. As a matter of fact the influence of gut microbiota on the risk of developing obesity in childhood is well known [81,82]. The gut microbiota maps individually obtained from children/adolescents after a MD regimen could be considered a new tool to evaluate MD effects on gut microbiota homeostasis [83], therefore on the risk of developing future diseases.

Physical activity, a non-sedentary lifestyle (reduced use of screens) [84], and a high parental educational level are related to healthier dietary styles in adolescents [48,64,66,67]. It has also been widely demonstrated how interventional nutritional programs can influence both diet and lifestyles [54,55]. However, studies evaluating the impact of distance-learning educational programs should increase in number.

In conclusion, an MD regimen seems associated with better pediatric outcomes in all age ranges. Implementing new strategies that allow the maintenance of an adequate dietary style and lifestyle in the long term remains one of today’s challenges.

## Data Availability

Not applicable.

## Supplementary Materials

The following supporting information can be downloaded at: https://www.mdpi.com/article/10.3390/children9060906/s1, Table S1: Missing data on the protective effect of the Mediterranean diet (MD) across different periods of life and future perspectives for research.
