# Supplementary material for: Mediterranean Diet in Developmental Age: A Narrative Review of Current Evidences and Research Gaps
Source: Children (Basel). 2022 Jun 16;9(6):906. doi: 10.3390/children9060906 (PMC9221965; doi:10.3390/children9060906)
Supplement: Supplementary file 1 [file children-09-00906-s001.zip › children-1744046-supplementary.pdf]

Table S1. Missing data on the protective effect of the Mediterranean diet (MD) across different periods of life and future perspectives for research.

**Period of life**	**Contrasting and missingevidence from previous studies**
Pregnancy	Intermediate quality of evidence associating maternal adherence to MD with intrauterine growth restriction, small-for-gestational-age new-borns and preterm delivery. Need for homogeneous definitions for MD, intrauterine growth restriction and preterm delivery.<br><br>A protective effect of maternal adherence to MD against malformations and congenital defects (including neural tube defects, congenital heart defects and gastroschisis) has to be investigated and established.<br><br>Conflicting evidence of a reduction in childhood asthma, allergies and related outcomes when mothers follow MD during pregnancy.
Lactation and weaning	Mother’s attention to MD is at the lowest during lactation. Re-sensitization programs should target this period and measure outcomes on children’s health.<br><br>No evidence suggests a positive correlation between mother’s dietary intake and atopic outcomes in children.
Preschool age (1-5 years)	After weaning, attention to children’s nutrition reduces. New strategies are needed to increase the role of expert professionals in guiding preschool children’s diet, reducing consumption of sugary drinks, snacks, proteins and sodium.<br><br>A protective role of MD in preventing celiac disease was established, and should be investigated also for other autoimmune diseases.
School age (6-12 years)	Despite its positive effect on health and prevention of several diseases (including asthma, gastrointestinal disorders, arterial hypertension and metabolic syndrome), adherence to MD in this age group is low (5%). New strategies targeting not only parents but also children and adolescents should be implemented.
Adolescence (10-19 years)	Adherence to MD is in Mediterranean countries, and particularly in rural locations and smaller cities. New interventions and nutritional re-education programs should focus on adolescents living in urban areas (including North of Italy).<br><br>It should be noted that, in this age group, MD might be ineffective if not combined with enough physical activity. Programs targeting adolescent should increase also their physical fitness to reduce effectively cardiovascular risk.<br><br>Evidence on a possible protective role of MD in diseases different from obesity and cardiovascular risk is lacking. Research should focus on the influence of an healthy diet, as the MD, on endocrinological, neuropsychological, gastrointestinal, and autoimmune disorders, also in the long term.
